# Zfp423 Promotes Adipogenic Differentiation of Bovine Stromal Vascular Cells

**DOI:** 10.1371/journal.pone.0047496

**Published:** 2012-10-10

**Authors:** Yan Huang, Arun Kr Das, Qi-Yuan Yang, Mei-Jun Zhu, Min Du

**Affiliations:** 1 Developmental Biology Group, Department of Animal Science, University of Wyoming, Laramie, Wyoming, United States of America; 2 Department of Animal Sciences, Washington State University, Pullman, Washington, United States of America; 3 School of Food Science, Washington State University, Pullman, Washington, United States of America; Mayo Clinic, United States of America

## Abstract

Intramuscular fat or marbling is critical for the palatability of beef. In mice, very recent studies show that adipocytes and fibroblasts share a common pool of progenitor cells, with Zinc finger protein 423 (Zfp423) as a key initiator of adipogenic differentiation. To evaluate the role of Zfp423 in intramuscular adipogenesis and marbling in beef cattle, we sampled beef muscle for separation of stromal vascular cells. These cells were immortalized with pCI neo-hEST2 and individual clones were selected by G418. A total of 288 clones (3×96 well plates) were isolated and induced to adipogenesis. The presence of adipocytes was assessed by Oil-Red-O staining. Three clones with high and low adipogenic potential respectively were selected for further analyses. In addition, fibro/adipogenic progenitor cells were selected using a surface marker, platelet derived growth factor receptor (PDGFR) α. The expression of Zfp423 was much higher (307.4±61.9%, *P*<0.05) in high adipogenic cells, while transforming growth factor (TGF)-β was higher (156.1±48.7%, *P*<0.05) in low adipogenic cells. Following adipogenic differentiation, the expression of peroxisome proliferator-activated receptor γ (PPARγ) and CCAAT/enhancer binding protein α (C/EBPα) were much higher (239.4±84.1% and 310.7±138.4%, respectively, *P*<0.05) in high adipogenic cells. Over-expression of Zfp423 in stromal vascular cells and cloned low adipogenic cells dramatically increased their adipogenic differentiation, accompanied with the inhibition of TGF-β expression. Zfp423 knockdown by shRNA in high adipogenic cells largely prevented their adipogenic differentiation. The differential regulation of Zfp423 and TGF-β between low and high adipogenic cells is associated with the DNA methylation in their promoters. In conclusion, data show that Zfp423 is a critical regulator of adipogenesis in stromal vascular cells of bovine muscle, and Zfp423 may provide a molecular target for enhancing intramuscular adipogenesis and marbling in beef cattle.

## Introduction

Marbling, or intramuscular fat, has been consistently identified as one of the top beef quality problems [1], [2]. Intramuscular adipocytes distribute throughout the perimysial connective tissue of skeletal muscle and are the major site for the deposition of intramuscular fat, which is essential for the eating quality of meat [3], [4]. In medicine, intramuscular adipocyte deposition so called fatty infiltration in skeletal muscle, is highly correlated with insulin resistance and type 2 diabetes [5]–[8].

It has been well established that stromal vascular cells are major sources of adipogenic cells in skeletal muscle [9]. Very recently in rodents, it observed that intramuscular adipocytes and fibroblasts share immediate common progenitor cells [10]. These progenitors are mainly located in the stromal vascular (SV) fraction of skeletal muscle and distinct from satellite cells [11], [12]. Enhancing adipogenesis of progenitor cells increases intramuscular fat (marbling), improving palatability of meat; on the other hand, less fibrogenic differentiation reduces intramuscular connective tissue, decreasing background toughness of meat. In combination, the eating quality of meat can be improved. To effectively enhance adipogenesis and reduce fibrogenesis, however, we must understand mechanisms regulating adipogenic and fibrogenic differentiation of progenitor cells in the SV fraction of skeletal muscle.

Adipogenesis is regulated by peroxisome proliferator-activated receptor γ (PPARγ) and CCAAT-enhancer-binding proteins (C/EBPs); their expression is critical for inducing the expression of genes specific to adipocytes [13], [14]. Recently, zinc-finger protein Zfp423 was identified as a transcriptional factor inducing PPARγ expression and the adipogenic commitment of progenitor cells [15]. Fibrogenesis is mainly mediated by transforming growth factor (TGF)-β signaling pathway [16], [17], which promotes fibrosis via activation of the Smad signaling pathway to initiate the transcription of TGFβ target genes [18]–[20], including fibronectin and type I collagen [21], [22].

To elucidate key mechanisms regulating adipogenic and fibrogenic differentiation of SV progenitor cells, we separated SV cells from bovine muscle, which were immortalized. Clones derived from individual SV cells were assessed for their adipogenic potential. Three clones with high and low adipogenic potential respectively were selected for further analyses. We hypothesized that the Zfp423 expression differ between cells with high and low adipogenic potential, and Zfp423 is a critical regulator of the adipogenic potential of bovine SV cells.

## Results

### SV cell selection

**Figure 1:**
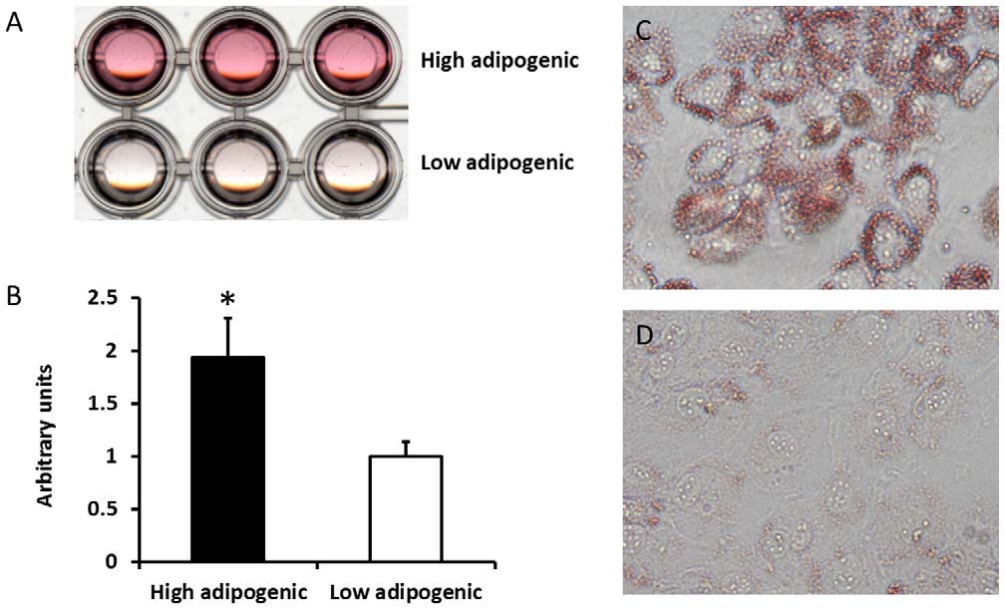
Adipogenic differention of cloned low and high adipogenic cells. A) Transmissive scanning of Oil-Red-O stained high and low adipogenic cells. B) Quantification of Oil-Red-O staining by absorbance measurement at 520 nm for high and low adipogenic cells. C) 100× magnified image of stained high adipogenic cells. D) 100× magnified image of stained low adipogenic cells. (*P<0.05, Mean ± SE; n = 3).

We prepared primary cultures of SV cells and transfected cells with pCI neo-hEST2 which contains a telomerase reverse transcriptase immortalizing cells without altering their phenotypes. Immortalized cells were seeded at very low density to obtain individual clones. A total of 288 clones (3×96 well plates) were isolated and induced adipogenic differentiation. Following 12 days of adipogenic differentiation, the presence of adipocytes was assessed by Oil-Red-O staining. Three clones with high and low adipogenic potential respectively were selected for further analyses (Fig. 1ACD). Cells without adipogenic differentiation were excluded. High adipogenic cells were stained much more intense (93.6±39.6%, *P*<0.05) than low adipogenic cells by Oil-Red-O (Fig. 1B).

### Adipogenic and fibrogenic mRNA expression

**Figure 2:**
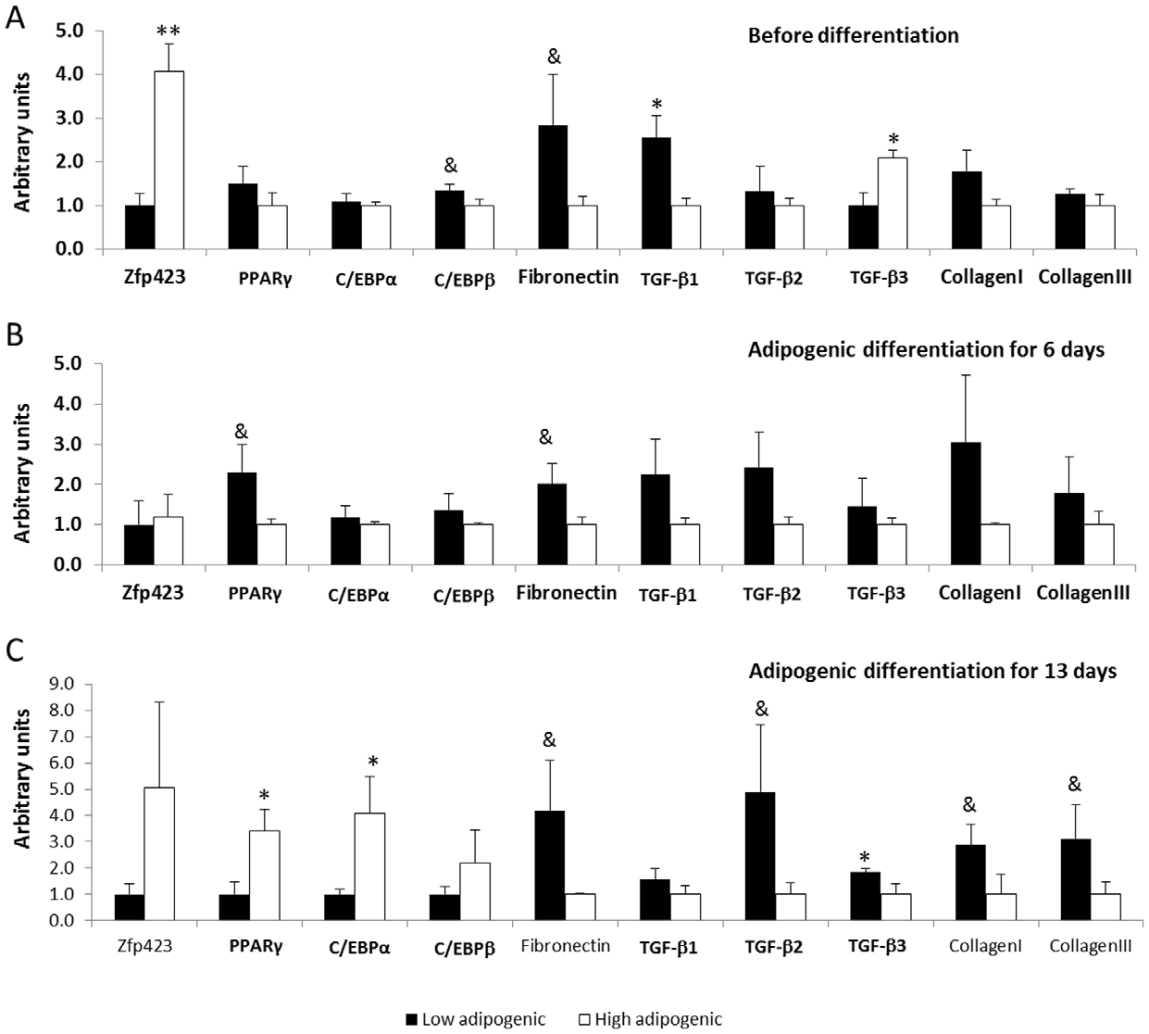
The mRNA expression of adipogenic and fibrogenic genes in low and high adiogenic cells. Cloned high and low adipogenic cells were induced adipogenic differentiation with a standard protocol. A) Gene expression before inducing differentiation. B) Gene expression at 6th day of adipogenic differentiation. C) Gene expression after 13 days of differentiation. (*P<0.05, ^&^ *P<*0.10; Mean ± SE; n = 3).

Between the two groups of cells, we checked mRNA expression of adipogenic and fibrogenic marker genes before inducing adipogenesis (Fig. 2). The expression of Zfp423 was much higher (307.4±61.9%, *P*<0.05) in high adipogenic cells, while TGF-β1 was higher (156.1±48.7%, *P*<0.05) in low adipogenic cells (Fig. 2A). There was no difference in the expression of PPARγ and C/EBPα, indicating that these cells were progenitor cells, not differentiated adipocytes.

After inducing adipogenic differentiation for 6 days, the difference in Zfp423 expression disappeared but PPARγ expression was higher in high adipogenic cells, showing enhanced adipogenic differentiation (Fig. 2B). Following 13 days of adipogenic differentiation, the expression of both PPARγ and C/EBPα was higher (239.4±84.1% and 310.7±138.4%, respectively, *P*<0.05) in high adipogenic cells than that of low adipogenic cells (Fig. 2C).

**Figure 3:**
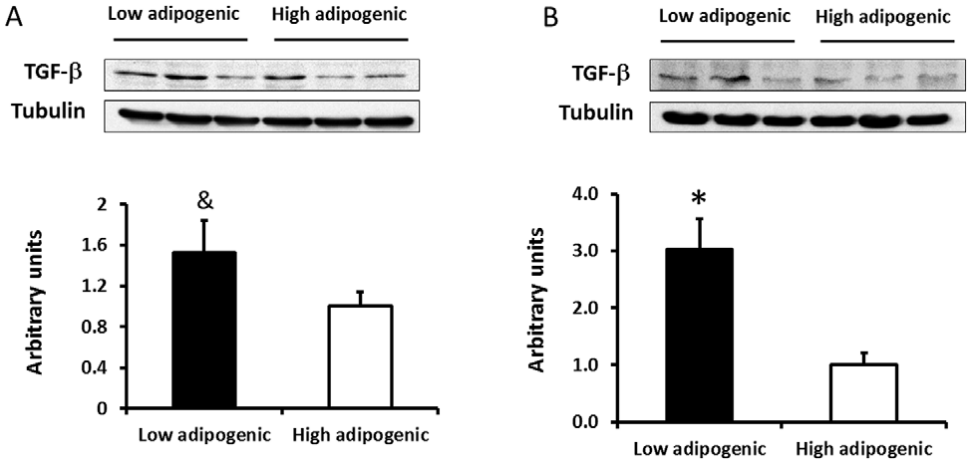
Transforming growth factor (TGF)-β protein content in low and high adipogenic cells. A) TGF-β content before adipogenic differentiation. B) TGF-β content after 13 days of adipogenic differentiation. (*P<0.05, ^&^ *P<*0.10; Mean ± SE; n = 3).

Western blotting was further conducted to determine the difference in TGF-β contents between these two groups of cells (Fig. 3). Low adipogenic cells tended to have higher TGF-β content (52.7±31.7%, *P*<0.10) (Fig. 3A). Following adipogenic differentiation, TGF-β content of low adipogenic cells was much higher (202.2±54.1%, *P*<0.05) than that of high adipogenic cells group (Fig. 3B), indicating that TGF-β production persisted in low adipogenic cells while decreased during differentiation in high adipogenic cells. We did not analyze the Zfp423 protein content because of lacking an appropriate antibody for bovine animals.

**Figure 4:**
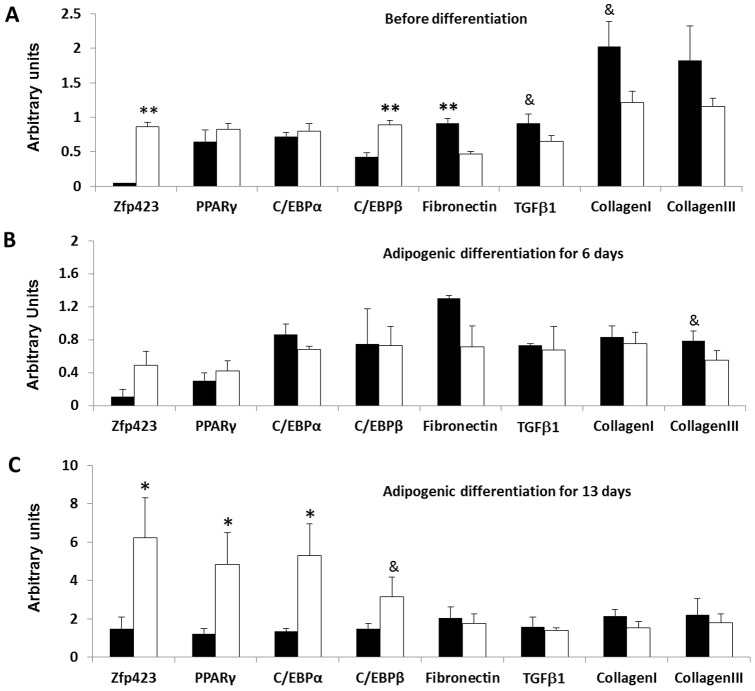
The mRNA expression of adipogenic and fibrogenic genes in sorted low and high adiogenic progenitor cells. Cloned high and low adipogenic cells were sorted by the surface marker, PDGFRα, and induced adipogenic differentiation with a standard protocol. A) Gene expression before inducing differentiation. B) Gene expression at 6th day of adipogenic differentiation. C) Gene expression after 13 days of differentiation. (*P<0.05, ^&^ *P<*0.10; Mean ± SE; n = 3).

To further confirm that such differential mRNA expression was due to the inherent difference in progenitor cells with high and low adipogenic potential, we sorted cloned cells by their expression of PDGFRα, a marker of fibro/adipogenic progenitor cells, and selected progenitor cell clones with low and high adipogenic potency. Above experiments were repeated in these cells. As shown in Fig. 4, the mRNA expression of low and high adipogenic progenitor cells was consistent with the clonally derived low and high adipogenic cells (Fig. 4), with higher expression of Zfp423 before differentiation and higher expression of both Zfp423 and PPARγ after differentiation compared to low adipogenic cells.

### Adipogenesis after over-expression of Zfp423

**Figure 5:**
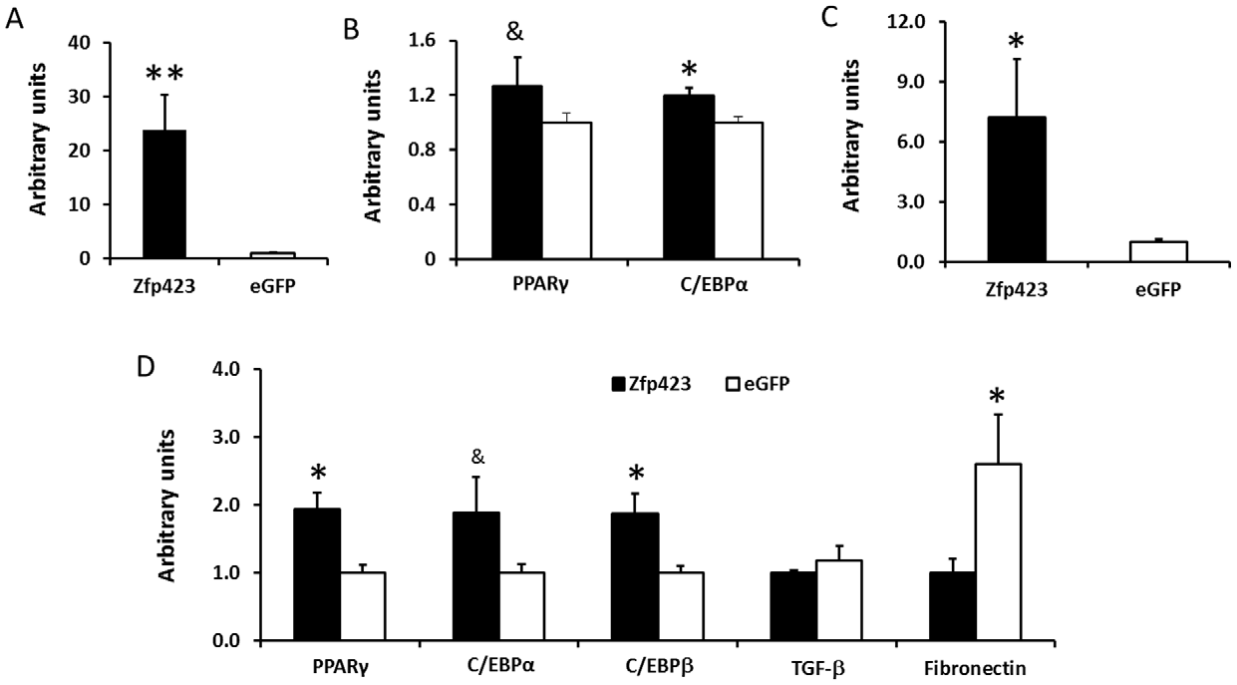
Adipogenic differentiation of stromal vascular cells over-expressing Zfp423. A) Zfp423 expression 3 days after transfection with a vector over-expressing Zfp423. B) expression of adipogenic marker genes PPARγ and C/EBPα after 3 days of Zfp423 over-expression. C) Zfp423 expression efficiency 6 days after transfection with a vector over-expressing Zfp423. D) expression of adipogenic marker genes PPARγ and C/EBPα after 6 days of Zfp423 over-expression. (*P<0.05, ^&^ *P<*0.10; Mean ± SE; n = 3).

To further define the role of Zfp423 in adipogenesis, we transfected bovine SV cells with a vector over-expressing Zfp-423 (Fig. 5A). After 3 days following transfection, Zfp423 over-expression significantly increased C/EBPα expression (19.6±5.7%, *P*<0.05), and PPARγ expression tended to be increased (26.6±21.3%, P<0.10) compared with cells transfected with a control plasmid carrying eGFP (Fig. 5B). After 6 days following Zfp423 transfection in SV cells, Zfp423 expression remained much higher in transfected cells (Fig. 5C), and the mRNA expression of PPARγ and C/EBPβ (93.3±24.8% and 87.1±52.8%, respectively, *P*<0.05) was dramatically increased, while the expression of C/EBPα tended to be increased (87.7±24.8%, *P*<0.10) in Zfp423 over-expressed cells. Meanwhile fibronectin expression was increased (160.2±73.3%, *P*<0.05) in low adipogenic cells (Fig. 5D).

**Figure 6:**
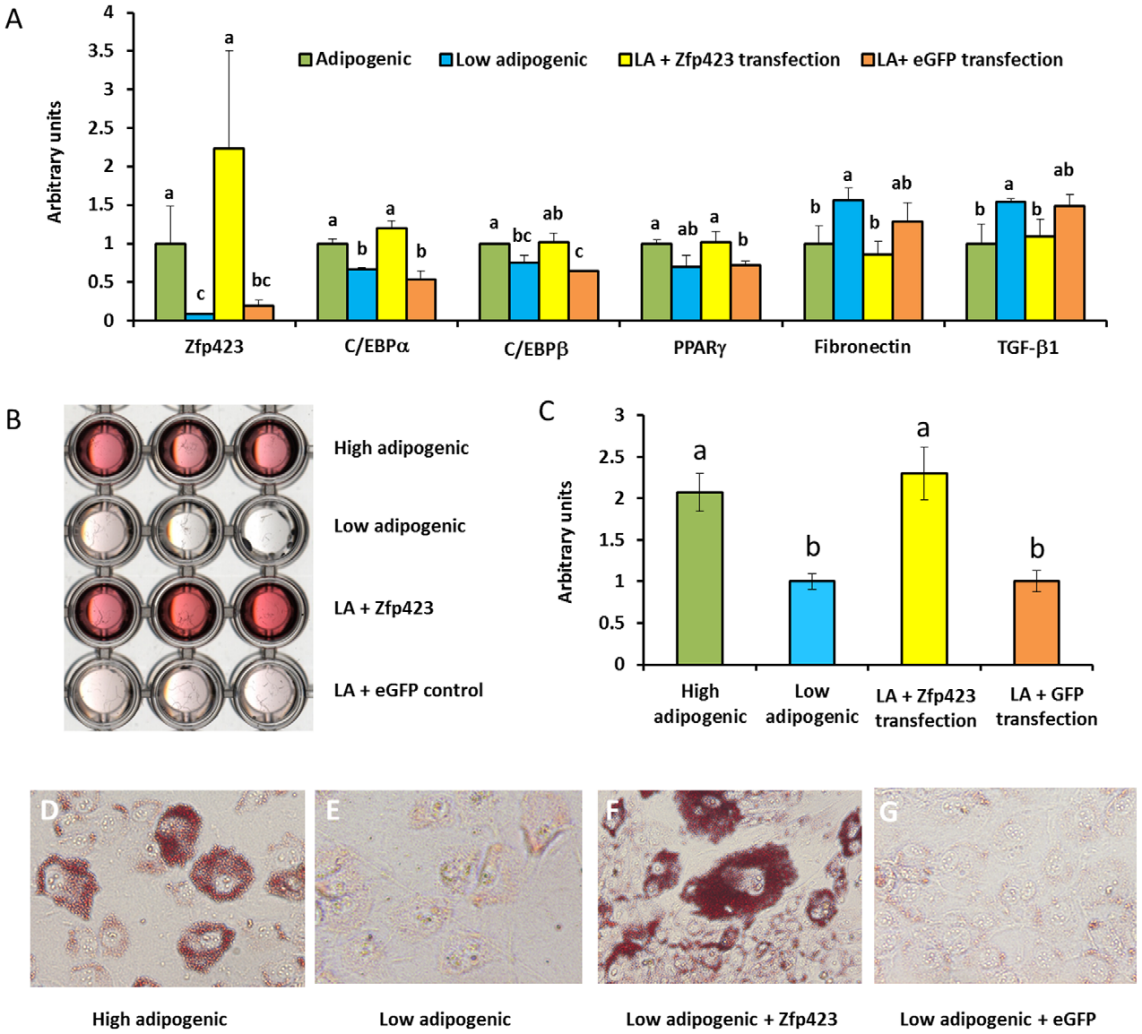
Adipogenic differentiation of cloned low adipogenic cells after Zfp423 transfection. A) adipogenic gene expression in control (eGFP transfected low adipogenic cells), Zfp423 transfected low adipogenic cells, and cells without transfection. B) Transmissive scanning of Oil-Red-O stained cells. C) Quantification of Oil-Red-O staining by absorbance measurement at 520 nm. D) 100× magnified image of high adipogenic cells. E) 100× magnified image of low adipogenic cells. F) 100× magnified image of Zfp423 transfected low adipogenic cells. G) 100× magnified image of eGFP tranfected low adipogenic cells. (^ab^Means bearing different superscripts indicate significant difference, *P<*0.05, Mean ± SE; n = 3).

Because Zfp423 expression was much higher in high adipogenic cells and Zfp423 is the earliest adipogenic differentiation marker, we speculated that Zfp423 might be responsible for the difference in the adipogenic potential between high and low adipogenic cells. To test this notion, we transfected low adipogenic cells with the Zfp423 expressing vector. Indeed, low adipogenic cells transfected with Zfp423 lifted their adipogenic potential to a level similar to that of high adipogenic cells (Fig. 6A). Interestingly, Zfp423 over-expression reduced the expression of TGFβ in low adipogenic cells, which indicate that TGFβ expression is at least partially regulated by Zfp423 (Fg. 6A).

The Oil-Red-O staining showed that those Zfp423 transfected low adipogenic cells form similar amounts of adipocytes as those high adipogenic cells (Fig. 6B), consistent with the qualification data (Fig. 6C) and images with high magnification (Fig. 6DEFG).

### Adipogenesis after knocking down Zfp423

**Figure 7:**
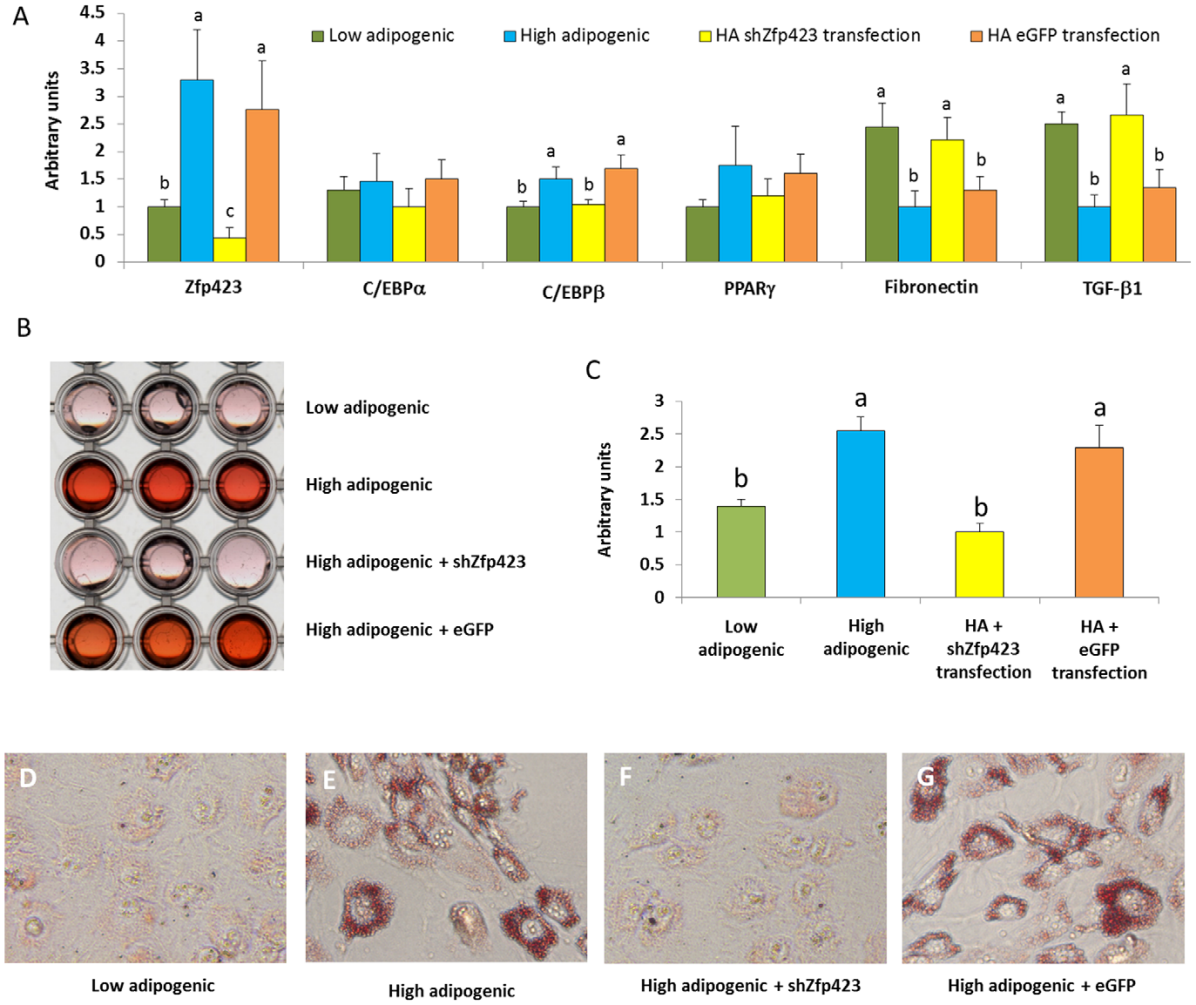
Adipogenic differentiation of cloned high adipogenic cells after shZfp423 knockdown. A) adipogenic gene expression in low adipogenic cells, high adipogenic cells, high adipogenic (HA) with shZfp423 to knockdown Zfp423, HA with eGFP (transfection control). B) Transmissive scanning of Oil-Red-O stained cells. C) Quantification of Oil-Red-O staining by absorbance measurement at 520 nm. DEFG) 100× magnified images showing Oil-Red-O stained adipocytes. (^ab^Means bearing different superscripts indicate significant difference, *P<*0.05, Mean ± SE; n = 3).

To determine if high adipogenic feature can be eliminated by inhibiting Zfp423 expression, shRNA against Zfp423 (shZfp423) were transfected in to high adipogenic cells. We observed that high adipogenic cells transfected with shZfp423 decreased the adipogenic potential to the similar level of low adipogenic cells (Fig. 7A).

The Oil-Red-O staining, qualification data, and cells images with high magnification showed that shZfp423 transfected high adipogenic cells decreased the accumulation of lipid droplets to the similar amount of low adipocytes generate (Fig. 7BCDEFG). These data show that Zfp423 is necessary and sufficient to regulate the adipogenic potential of progenitor cells, clearly demonstrating the importance of Zfp423 in the adipogenic commitment of bovine cells.

### Methylated DNA immunoprecipitation (MeDIP)

**Figure 8:**
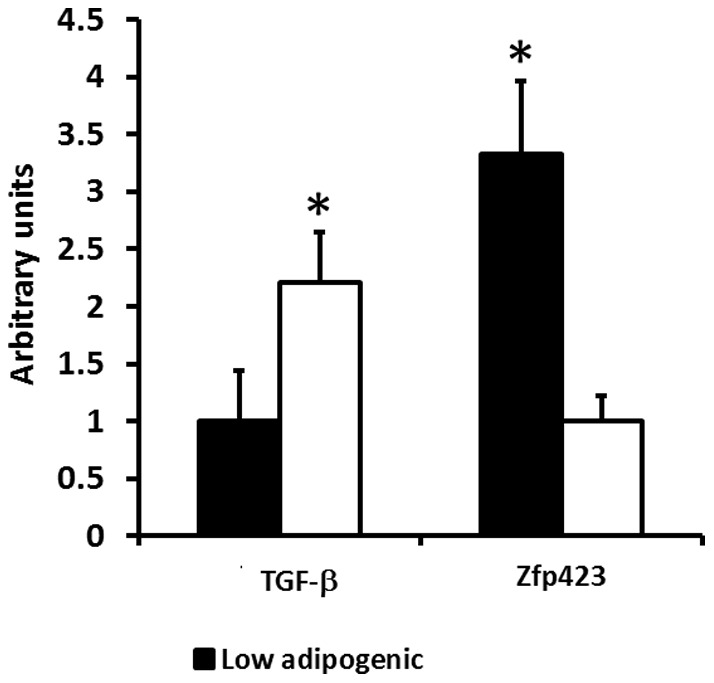
MeDIP analysis of bovine TGFβ and Zfp423. (*P<0.05, Mean ± SE; n = 3).

Because those low and high adipogenic cells were separated from a single bovine animal, these cells have identical genomic sequences. Therefore, their differential adipogenic potential should be due to epigenetic modifications. Because these cloned cells have been proliferated for many cell generations, only stable DNA methylation was expected to be kept, which affects progenitor cell differentiation. To further test whether DNA methylation is involved in the differential expression of Zfp423 and TGFβ between low and high adipogenic cells, we first analyzed the content of GC sites in the promoter regions of these two genes. To our excitement, both promoters have exceptionally high contents of GC sites and several GC islands. To test whether there is a difference in DNA methylation in the promoters of Zfp423 and TGF-β between low and high adipogenic cells, we used MeDIP (Fig. 8). Higher density of DNA methylation was detected in the TGF-β promoter of high adipogenic cells (120.9±43.9%, P<0.05) compared with low adipogenic group. By contrast, low adipogenic cells had higher density of DNA methylation in the Zfp423 promoter when compared to high adipogenic cells (233.0±63.1%, P<0.05).

**Table 1:** List of bovine primers.

1Primers	Forward sequence	Reverse sequence
RtPCR:		
Zfp423	5′- GGATTCCTCCGTGACAGCA -3′	5′- TCGTCCTCATTCCTCTCCTCT -3′
PPARγ	5′- TGGAGACCGCCCAGGTTTGC -3′	5′- AGCTGGGAGGACTCGGGGTG -3′
C/EBPα	5′- TGCGCAAGAGCCGGGACAAG -3′	5′- ACCAGGGAGCTCTCGGGCAG -3′
C/EBPβ	5′- CGGGCAGCACCACGACTTCC -3′	5′- CCCCAGTCGGCCCAGACTCA -3′
Fibronectin	5′- GCGTGTCACCTGGGCTCCAC-3′	5′- CGGTGCCGGGCAGGAGATT-3′
TGF-β1	5′- AGCCAGGGGGATGTGCCA -3′	5′- TAGCACGCGGGTGACCTCCT -3′
TGF-β2	5′-CATCTGGTCACGGTCGCGC-3′	5′-GGGACCTCCTCGGGTTCGGG-3′
TGF-β3	5′-AACGCAGGTCCTGGGGGTCA-3′	5′-CTCCAGTCGTGTGCGCCCTG-3′
Collagen type I	5′- CCACCCCAGCCGCAAAGAGT-3′	5′- ACGCAGGTGACTGGTGGGATGTC-3′
Collagen type III	5′- GGCCCCCTGGAAAGGACGGA-3′	5′- CCCCGCCAGCACCACAACAT-3′
18S	5′- CCTGCGGCTTAATTTGACTC -3′	5′- AACTAAGAACGGCCATGCAC -3′
MeDIP:		
Zfp423	5′- TCCTCAGATGGGTTCATTGTGAGCT-3′	5′- TCCTCCTGCCTCTCGGAAACTTT-3′
TGF-β1	5′- CACCGGCATCATGCACACG -3′	5′- AAGGGCCGTGTTTCCACAGTCC -3′

## Discussion

According to the past surveys of beef producers by National Cattlemen's Beef Association, marbling and tenderness were consistently identified as the top beef quality problems [1], [2]. Marbling becomes a top quality problem because of the selection for high lean growth, which results in the overall reduction in fat accumulation, including intramuscular fat (marbling) [23]. Hence, to improve the eating quality of meat, we have to increase the marbling of beef and other meats. Intramuscular adipocytes provide sites for triglyceride deposition. Very recent studies show that intramuscular adipocytes and fibroblasts are developed from common progenitor cells in the SV faction of skeletal muscle [10]–[12]. Up to now, however, mechanisms regulating the early commitment of intramuscular adipogenic differentiation remain elusive, especially in farm animals.

Here, we separated SV cells which contained a mixture of fibroblasts, pre-adipocytes and progenitor cells, without differentiated adipocytes which were removed through centrifuge. Those SV cells were immortalized by over-expression of telomerase reverse transcriptase, and individual cell derived clones were isolated and induced adipogenesis. Fibroblasts were excluded by their lack of adipogenic differentiation. Therefore, those three cells with low and high adipogenic differentiation selected for the current study likely represent progenitor cells or cells at the early stage of adipogenic or fibrogenic differentiation, ideal for studying transcription factors or mediators committing these cells to adipogenesis.

TGF-β signaling is critical for fibrogenic differentiation, which propels the synthesis of collagen and extracellular matrix [16], [17], [24]. Interestingly, the expression of TGF-β was higher in low adipogenic cells, indicating that these cells are likely positioned for fibrogenic differentiation.

Adipogenesis is regulated by PPARγ and C/EBPα [13], [14]. PPARγ expression is necessary for adipogenesis and is a marker for adipogenic cells [25], [26]. PPARγ and C/EBPα enforce each other and synergize to induce the expression of genes specific to adipocytes [27]. There was no difference in the expression of PPARγ and C/EBPα between high and low adipogenic cells before inducing adipogenic differentiation, which indicates that both high and low adipogenic cells were progenitor cells or cells at the very early stage of differentiation since increase in PPARγ expression is necessary for adipogenic differentiation. After 13 days of differentiation, however, the expression of both PPARγ and C/EBPα were dramatically higher in high adipogenic cells, consistent with their high adipogenic potential.

To further confirm that these cells represent progenitor cells, we sorted these cells using a surface marker of fibro/adipogenic progenitor cells, platelet derived growth factor receptor (PDGFR) α. [10], [11], and analyzed the expression of adipogenic and fibrogenic markers before and after adipogenic differentiation as above. Very consistently, the expression of these markers was very similar to those high and low adipogenic cells without sorting, further confirming that these cloned cells were progenitor cells or cells at the very early stage of commitment.

Zfp423 is a recently identified transcription factor key to the early commitment of adipogenesis [15]. We found that the Zfp423 expression was higher in high adipogenic cells than that of low adopogenic cells, which is in agreement with a very recent report describing that Zfp423 is a preadipocyte determination factor in mice [28]. These data prompted us to hypothesize that the differential expression of Zfp423 is mainly responsible for the difference in adipogenic potential between high and low adipogenic cells. To test this, we over-expressed Zfp423 in bovine SV cells, and we observed that the expression of PPARγ, C/EBPα and C/EBPβ were dramatically increased. Since PPARγ and C/EBPα are critical transcription factors responsible for adipogenesis [29], Zfp423 may promote the expression of these two factors to induce adipogenesis of SV cells [15]. Because the SV cells contain a mixture of cells, to further define the role of Zfp423 in bovine adipogenesis, we over-expressed Zfp423 in low adipogenic cells, which pronouncedly enhanced their adipogenic potential to a level comparable to high adipogenic cells, clearly showing that Zfp423 is a critical factor mainly responsible for the difference in adipogenic potential between high and low adipogenic cells of bovine animals. Interestingly, enhanced Zfp423 expression reduced the expression of TGF-β, indicating that TGF-β is located down-stream of Zfp423. To conclusively establish the link between Zfp423 and adipogenesis, we further knockdown Zfp423 expression in high adipogenic cells, and as expected, adipogenesis in high adipogenic cells was dramatically reduced due to Zfp423 knockdown, clearly showing the regulatory role of Zfp423 in adipogenesis.

We next explored the mechanisms responsible for the differential expression of Zfp423 and TGF-β between low and high adipogenic cells. Because these cells were derived from a single bovine animal and have the same genomic DNA, the differential expression of Zfp423 and TGF-β are likely due to epigenetic modifications. Indeed, we observed rich GC sites in the promoter of Zfp423 and TGF-β genes, which indicates that DNA methylation is very likely to be the key factor regulating Zfp423 and TGF-β expression and, thus, the early commitment of adipogenesis. DNA methylation leads to permanent inhibition of gene expression. As expected, our MeDIP results show that the DNA methylation of the Zfp423 promoter was higher in low adipogenic cells, in line with the low level of Zfp423 expression in low adipogenic cells. On the other hand, the DNA methylation of TGF-β was higher in high adipogenic cells, consistent with the lower TGF-β expression in these cells and their high adipogenic potential. The remaining question is what regulates DNA methylation in the promoters of Zfp423 and TGF-β? A question we are planning to address in our subsequent studies.

In summary, data show that Zfp423 is a critical regulator of adipogenesis in bovine SV cells. Zfp423 enhances the expression of PPARγ and C/EBPα, while inhibiting fibrogenic differentiation via the inhibition of TGF-β expression. In this study, we only evaluate the role of Zfp423 in intramuscular adipogenesis, and adipogenic differentiation in other depots of beef cattle likely follow the same pattern. Indeed, Zfp423 was first demonstrated to be critical for adipogenic commitment in subcutaneous fat depot [15]. The generic role of Zfp423 in adipogenesis raises a question about how to target the intramuscular adipogenesis. Because adipocytes are first formed in visceral fat depots, followed by subcutaneous, intermuscular and intramuscular fat depots, it provides an opportunity to preferentially enhance intramuscular adipogenesis over other fat depots [30]. Administration of specific drugs or other compounds targeting Zfp423 expression to enhance adipogenesis during the early weaning stage to about 250 days of age, “a mabling window” [31]–[35], is expected to specifically enhance intramuscular adipogenesis, which provide sites for lipid accumulation during the “fattening” stage, specifically enhancing marbling [30]. Therefore, Zfp423 may provide a molecular target for enhancing intramuscular adipogenesis and marbling in beef cattle.

## Materials and Methods

### SV cell separation and immortalization

The *Sternocleidomastoid* muscle was sampled from the carcass of an Angus heifer (20 months of age) immediately after slaughter in the Meat Lab of the University of Wyoming. The heifer was slaughtered according to the USDA regulation. The isolation and culturing of primary SV cells was carried out as previously described [36]. Briefly, perimysial connective tissue was separated and minced with a dissecting scissor. The minced tissue was rinsed in pre-cooled PBS, then transferred to PBS containing 1 mg/ml collagenase type I. The mixture was digested for 40 min at 37°C with constant agitation. Then, the mixture was filtered through a 250 µm strainer, and filtrate was centrifuged at 400× g for 5 min. The pellet containing SV cells was resuspended in DMEM +10% FBS +1% antibiotics cocktail (10,000 IU/ml Penicillin +10,000 µg/ml Streptomycin +25 µg/ml Amphotericin B) and incubated at 37°C in 5% CO_2_. These cells were then immortalized with a mammalian expression vector containing telomerase reverse transcriptase (pCI neo-hEST2, Addgene, Cambridge, MA). Individual clones with stable transfection were selected by G418 (400 µg/ml).

### Adipogenic differentiation of cloned SV cells

A total of 288 clones (3×96 well plates) were isolated and induced to adipogenic differentation with a standard cocktail containing insulin (1 μg/ml), dexamethazone (0.1 μg/ml), isobutylmethylxanthine (27.8 μg/ml), and troglitazone (10 μM) for 6 days and, then, insulin only (1 μg/ml) for 6 more days. The presence of adipocytes was assessed by Oil-Red-O staining as described previously [37]. The presence of Oil-Red O dye in adipocytes was further quantified by measuring the optical absorbance at 520 nm. Three clones with the high and low adipogenic potential were selected for further analyses. Clones without adipogenic differentiation were excluded.

### Sorting of fibro/adipogenic progenitor cells

Using platelet drived growth factor receptor α (PDGFRα) as a surface marker, cloned SV cells were subjected to sort with MACS magnetic sorter (Miltenyi Biotec Inc., Auburn, CA) [11]. The antibody against PDGFRα was purchased from Cell Signaling (Cat#: 3174, Danvers, MA).

### Antibodies

Antibodies against tubulin (no. 2128) and TGF-β (no. 3711) were purchased from Cell Signaling (Danvers, MA).

### Western blot analysis

Western blotting was conducted as previously described [38] using an Odyssey Infrared Imaging System (LI-COR Biosciences, Lincoln, NE). Density of bands was quantified and then normalized according to the tubulin content.

### Real-time quantitative PCR (RT-PCR)

Total mRNA was extracted from muscle using TRI reagent (Sigma, St. Louis, MO) and reverse transcribed into cDNA by using a kit (Qiagen, Valencia, CA). RT-PCR was performed using an iQ5 RT-PCR detection system (Bio-Rad Laboratories, Hercules, CA). A SYBR Green RT-PCR kit from Bio-Rad Laboratories (Hercules, CA) was used together with the *Bos taurus* primers listed in Table 1. Each reaction yielded amplicons between 80 and 200 bp. PCR conditions were as follows: 20 s at 95°C, 20 s at 56°C, and 20 s at 72°C for 35 cycles. After amplification, a melting curve (0.01 C/s) was used to confirm product purity, and the PCR products were electrophoresed to confirm the targeted sizes. Results are expressed relative to 18S using the ΔΔCt method [39].

### Plasmid and transfection

Plasmid pMSCVFLAG-ZFP423 (Cat. 24764) was obtained from Addgene (Cambridge, MA). Plasmid transfection was performed using PolyJet *in vitro* DNA transfection reagent (SignaGen Laboratory, Ijamsville, MD) according to the manufacturer's instructions. Transfections were carried out when cells reached 95% confluence, using a 1∶3 ratio of DNA (μg): PolyJet (μl); medium was switched to DMEM medium containing 10% FBS and 1% antibiotics 12 hr following transfection.

### MeDIP

The procedure was conducted as previously described [40], [41]. Briefly, bovine cloned cells were cross-linked with 1% formaldehyde in PBS for 10 min at room temperature. Glycine was added to the final concentration of 0.125 M and the mixture incubated for 5 min to stop the reaction. Cross-linked cells were washed with PBS, lysed in 250 ml SDS lysis buffer and sonicated. Sheared DNA was denatured at 95°C in order to yield single stranded DNA fragments. Samples were pre-cleared with 60 µl protein A Sepharose beads and 10 µl supernatant per sample was saved as the total input. Samples were immunoprecipitated overnight at 4°C with antibodies against 5-methylcytidine (anti-5mC, Abd Serotec, Raleigh, NC) under shaking. The chromatin: antibody complex was incubated with Sepharose beads for 2 h at 4°C before washing and DNA elution, and precipitated DNA fragments were purified. The extracted DNA was used for PCR amplification of Zfp423 and TGF-β promoters using an iQ5 RT-PCR detection system (Bio-Rad Laboratories, Hercules, CA). A SYBR Green RT-PCR kit from Bio-Rad Laboratories (Hercules, CA) together with the *Bos taurus* primers for MeDIP listed in Table 1 were used. After amplification, a melting curve (0.01 C/s) was used to confirm product purity, and the PCR products were electrophoresed to confirm the targeted sizes.

### Statistical analyses

Data were analyzed as a complete randomized design using general linear model (GLM) of SAS. Differences in mean values were compared by Tukey's multiple comparison test, and means ± SE was reported. Statistical significance was considered as *P*<0.05.
